# Evolutionary interpretations of mycobacteriophage biodiversity and host-range through the analysis of codon usage bias

**DOI:** 10.1099/mgen.0.000079

**Published:** 2016-10-21

**Authors:** Lauren A. Esposito, Swati Gupta, Fraida Streiter, Ashley Prasad, John J. Dennehy

**Affiliations:** ^1^​ Biology Department, Queens College, Queens, NY 11367, USA; ^2^​ Biology PhD Program, The Graduate Center of the City University of New York, New York, NY 10016, USA

**Keywords:** bacteriophages, codon bias, host range, mycobacteria, UPGMA clustering, viral tRNAs

## Abstract

In an genomics course sponsored by the Howard Hughes Medical Institute (HHMI), undergraduate students have isolated and sequenced the genomes of more than 1,150 mycobacteriophages, creating the largest database of sequenced bacteriophages able to infect a single host, *Mycobacterium smegmatis*, a soil bacterium. Genomic analysis indicates that these mycobacteriophages can be grouped into 26 clusters based on genetic similarity. These clusters span a continuum of genetic diversity, with extensive genomic mosaicism among phages in different clusters. However, little is known regarding the primary hosts of these mycobacteriophages in their natural habitats, nor of their broader host ranges. As such, it is possible that the primary host of many newly isolated mycobacteriophages is not *M. smegmatis*, but instead a range of closely related bacterial species. However, determining mycobacteriophage host range presents difficulties associated with mycobacterial cultivability, pathogenicity and growth. Another way to gain insight into mycobacteriophage host range and ecology is through bioinformatic analysis of their genomic sequences. To this end, we examined the correlations between the codon usage biases of 199 different mycobacteriophages and those of several fully sequenced mycobacterial species in order to gain insight into the natural host range of these mycobacteriophages. We find that UPGMA clustering tends to match, but not consistently, clustering by shared nucleotide sequence identify. In addition, analysis of GC content, tRNA usage and correlations between mycobacteriophage and mycobacterial codon usage bias suggests that the preferred host of many clustered mycobacteriophages is not *M. smegmatis* but other, as yet unknown, members of the mycobacteria complex or closely allied bacterial species.

## Data Summary

All genomic sequence data analyzed in this study were downloaded from NCBI GenBank via links provided at phagesdb.org. The GenBank sequence accession numbers are provided in Tables S1 and S2 (available in the online Supplementary Material) for bacteriophage and Mycobacterial genome sequences respectively.

## Impact Statement

Through a course in bacteriophage discovery and genomics, thousands of undergraduate students isolated and sequenced the genomes of bacterial viruses (bacteriophages) able to infect the bacterial host, *Mycobacterium smegmatis*, thus creating the largest database of bacteriophages able to infect a single host type. However, little is known about the genetic organization of these phages or of their natural hosts in the wild. Here we use bioinformatic analyses to identify relationships among these phages and sequenced mycobacterial species. Based on our bioinformatic analyses, we report that *M. smegmatis* is unlikely to be the preferred host for many of these newly isolated bacteriophages. Instead we suggest that many isolated mycobacteriophages infect similar, but as yet unknown, mycobacterial species or have recently gained the ability to infect *Mycobacteria*.

## Introduction

Bacteriophages are the most populous organisms in the biosphere, but surprisingly little is known about their natural diversity and host ranges (Dennehy, 2014). One of the best-studied groups of phages are the mycobacteriophages, which infect mycobacterial hosts such as *Mycobacterium tuberculosis* and *Mycobacterium smegmatis*. To date, students participating in the Howard Hughes Medical Institute (HHMI)-sponsored Science Education Alliance–Phage Hunters Advancing Genomics and Evolutionary Science (SEA-PHAGES) initiative (seaphages.org) have isolated almost 7000 mycobacteriophages from soil samples using the host *M. smegmatis* (phagesdb.org). Of these, more than 1150 mycobacteriophage genomes have been fully sequenced and annotated for open reading frames (ORFs), tRNA genes and other features (Pope *et al.*, 2015). One surprising finding is that despite having the ability to infect the same host many mycobacteriophages share little or no genetic similarity (Pope *et al.*, 2015; Brüssow & Hendrix, 2002). Moreover, extensive genomic mosaicism makes it impossible to determine the phylogeny of mycobacteriophages (Pedulla *et al.*, 2003; Cresawn *et al.*, 2011). Instead mycobacteriophages exist in constellations of closely related phages, termed clusters, constituting a continuous spectrum of genetic diversity (Pope *et al.*, 2015; Grose & Casjens, 2014).

Despite the expanding knowledge of mycobacteriophage diversity and genetic content, little is known about their life history and ecology. Some of these newly isolated phages infect and form plaques on *M. tuberculosis* and *Mycobacterium bovis* (Rybniker *et al.*, 2006; Jacobs-Sera *et al.*, 2012), but we have little insight into the broader host ranges of these phages or of their preferred hosts in the wild. The SEA-PHAGES ecological data is mainly limited to the geographic coordinates of isolation, the date of isolation, and occasionally the uncurated discovery notes. As such, analysis of mycobacteriophage genomic sequences may be one of the best ways of acquiring ecological and evolutionary insight.

In this study, we analyzed the codon usage patterns and DNA GC content of 199 different mycobacteriophage genomes to determine if codon usage and DNA GC content patterns suggest evolutionary relationships or possible preferred hosts. Codon usage bias refers to the differences in the frequency of use of synonymous codons during protein synthesis. Despite having multiple synonymous codons for a given amino acid, organisms do not use these codons randomly or at equal frequencies (Sharp & Li, 1987; Hilterbrand *et al.*, 2012), suggesting that codon usage bias may affect organismal fitness and/or function (Kudla *et al.*, 2009; Parmley & Hurst, 2007). Since phages do not encode ribosomes, they are entirely dependent on their host’s translational machinery for replication. Efficient translation of a phage's proteins within a host is optimized by the phage's ability to match the codon usage patterns of their hosts (Carbone, 2008; Lucks *et al.*, 2008). Hence we expect a correlation between the codon usage patterns of the phage and its host. An exception to this pattern may occur when phages encode their own tRNAs (Bailly-Bechet *et al.*, 2007; Chithambaram *et al.*, 2014). Consequently, the mycobacteriophage codon usage patterns will most closely resemble that of the preferred host, except in cases where the phage encodes its own tRNA for a particular amino acid.

## Methods

### Mycobacteriophage genomic analysis.

Genome sequence data was obtained from the SEA-PHAGES initiative (phagesdb.com) for phages that were previously clustered into groups according to their nucleotide similarity (Pope *et al.*, 2015). Two genomes are placed in the same cluster if: (1) dot plot sequence similarity is >50 % of the smaller of the two genomes; (2) average nucleotide identity is >70 %; (3) the bioinformatics program Splitstree (Huson & Bryant, 2006) assigns the two genomes to a clearly defined group; and (4) the two genomes show a high degree of genome module similarity based on pairwise sequence alignments (Hatfull *et al.*, 2010). Phages that do not meet all of these criteria are not assigned to a cluster and are termed ‘singletons’ because they have no close relatives. The SEA-PHAGES initiative identified 26 different mycobacteriophage clusters and numerous subclusters and singletons (phagesdb.com) (Pope *et al.*, 2015). Members of a cluster tend to share genome architectures in addition to sequence similarities, and have similar genome lengths and numbers of genes per genome (Pope *et al.*, 2015).

Another layer of genomic analysis is the assignment of genes into ‘phams’, or groups of closely related sequences, using the program Phamerator (Cresawn *et al.*, 2011). Two genes share a pham if the amino acid sequence identity given by ClustalW alignments is >32.5 % and the if BlastP E-value is <10^–50^ (Cresawn *et al.*, 2011). As of he time of writing, the total number of mycobacteriophage phams assigned is more than 21 000, which suggests a tremendous wealth of biologically novel genes (phagesdb.com).

### Selection of mycobacteriophages and mycobacterial species.

Mycobacteriophages were selected for this study based on the availability of a fully annotated genome in the NCBI GenBank as of June 2013. In most clusters, all fully sequenced mycobacteriophages available were selected for further study. However in the A cluster, some subclusters contained more phages than many of the other clusters. Therefore, in order to avoid redundancy and biased results, 10 phages from each of the A1 and A4 subclusters of the A cluster were selected at random. A total of 199 complete mycobacteriophage genomes were downloaded from NCBI GenBank (Table S1). Seven species of the genus *Mycobacterium*, *M. smegmatis* mc^2^155*, M. bovis* BCG*, M. tuberculosis* H3R7v*, Mycobacterium avium* K-10, *Mycobacterium leprae* TN, *Mycobacterium ulcerans* AGY99 and *Mycobacterium abscessus* bolletii 50594, were selected based on previously known infectivity patterns with the selected mycobacteriophages (Rybniker *et al.*, 2006; Jacobs-Sera *et al.*, 2012) and their full genomes were downloaded from NCBI GenBank (Table S2).

### Analysis of codon ssage bias.

The relative synonymous codon usage order (RSCU) is the ratio of the observed frequency of codons to the frequency expected if all synonymous codons were used equally (Sharp & Li, 1987). RSCU values were calculated for all mycobacteriophage and mycobacterial genomes using the program mega 6.1 (megasoftware.net) (Tamura *et al.*, 2013). The RSCU values for all bacterial genomes were obtained from a previously reported analysis on the codon bias database (CBDB; cbdb.info) (Hilterbrand *et al.*, 2012). Synonymous codon usage order (SCUO) is a measure determining the synonymous codon usage bias within and across genomes (Wan *et al.*, 2004, 2006). SCUO is a newer method of analyzing codon usage bias, and is based on Shannon's information theory. We selected this method for several reasons. Since we have no a priori knowledge of bacteriophage gene expression levels, and do not have validated reference genomes, codon analysis methods based on reference genomes, such as the Codon Adaption Index (CAI), may not provide a robust analysis. Second, SCUO takes genome GC composition into account, which may be more appropriate given that mycobacteriophage and mycobacterial genomes are highly GC-biased. Furthermore, since our comparisons are mainly between genomes, including comparisons between bacteriophage and bacteria genomes, rather than among genes, we chose SCUO since it is considered to be a more robust method for between-genome comparisons. The SCUO for each genome was calculated using the program INCA 2.1 (bioinfo.hr/research/inca/).

### tRNA abundance and synonymous codon usage order.

**Fig. 1.**
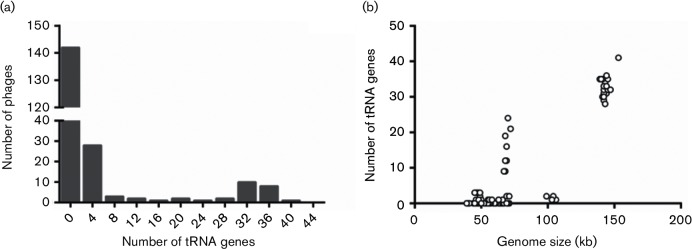
Histogram of tRNA-encoding genes found in the 199 mycobacteriophages analyzed in this study.

**Fig. 2.**
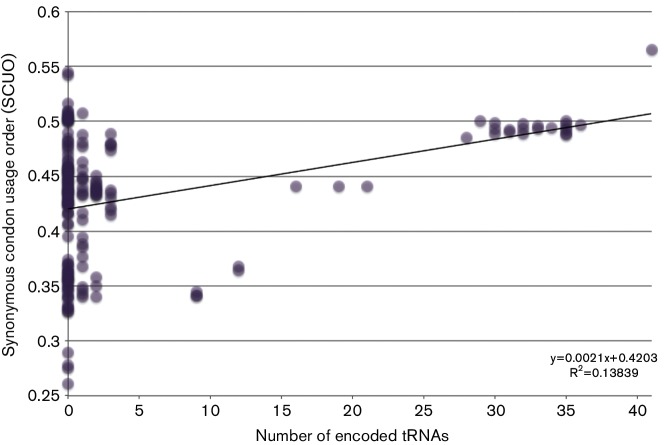
The synonymous codon usage order (SCUO) was calculated for each genome. SCUO values for each mycobacteriophage were compared with the quantity of encoded tRNA genes. tRNA abundance does not predict SCUO. However, if a phage does have tRNA genes, it tends to have a more biased genome.

Genes encoding tRNAs are often found in mycobacteriophage genomes (Bailly-Bechet *et al.*, 2007) (Figs 1, 2). Bacteriophages use their host’s translational machinery to reproduce, which limits successful propagation to the tRNA pool found in the host (Bailly-Bechet *et al.*, 2007; Plotkin & Kudla, 2011). Because of this, bacteriophages encoding their own tRNAs are predicted to have higher codon usage biases than bacteriophages that do not encode their own tRNAs. We used the software tRNAscan-SE 1.21 to identify tRNA genes in mycobacteriophage genomes downloaded from NCBI GenBank (Lowe *et al.*, 1997; Schattner *et al.* 2005) (lowelab.ucsc.edu/tRNAscan-SE). SCUOs were determined for genomes that encode and do not encode their own tRNAs in order to determine if tRNA prevalence is correlated with codon usage bias.

### Cluster analysis based on phage and host RSCU values.

Cluster analysis has been used to study the patterns of codon usage bias of genes within a genome, as well as across organisms (Sharp & Li, 1987). The RSCU for each bacteriophage was compared with those of all other bacteriophages using Pearson's correlation coefficient. A distance matrix was constructed, where the distance value (*d*)=(1−*r*)×100 where *r* is the Pearson coefficient. In this study, we used the software dendroUPGMA to construct unweighted pair group method with arithmetic mean (UPGMA) dendrograms to cluster all genomes and structural proteins according to their RSCU values (Garcia-Vallvé *et al.*, 1999) (genomes.urv.cat/UPGMA). From this point forward, we will refer to the above-mentioned clusters as UPGMA clusters or nodes in order to distinguish these from the SEA-PHAGES clusters and subclusters created on the basis of genetic similarities.

## Results and Discussion

### Mycobacteriophage GC content and comparisons with mycobacterial GC content

Despite a universal bias towards GC→AT mutations (Hildebrand *et al.*, 2010; Hershberg & Petrov, 2010; Ran *et al.*, 2014), genome GC content ranges widely among in prokaryotes (Foerstner *et al.*, 2005; Bohlin *et al.*, 2010, Bentley & Parkhill, 2004), and some organisms such as the *Mycobacteria*, have high DNA GC content. Studies have shown that DNA GC content is correlated with genome length (Mitchell, 2007; Musto *et al.*, 2006; Pedulla *et al.*, 2003), phylogeny (Hershberg & Petrov, 2010) and ecological and environmental factors (Foerstner *et al.*, 2005). The *Actinobacteria*, the phylum in which the *Mycobacteria* are classified, are known for their high DNA GC content. This high DNA GC content may reflect the complexities of the soil habitat characteristic of many actinobacterial species (Foerstner *et al.*, 2005; Tringe *et al.*, 2005).

While the high DNA GC content of the mycobacteriophages with small genomes seems at odds with the correlation between GC content and genome size, this trait is likely to be a result of correlations between the mycobacteriophages and their high-DNA-GC-content mycobacterial hosts (e.g. *M. smegmatis* DNA GC content: 68 mol%) (Bahir *et al.*, 2009; Andersson & Sharp, 1996; Xia & Yuen, 2005). Since DNA GC content constrains codon usage and, therefore, may affect translational efficiency, it is expected that virus DNA GC content will match that of their hosts (Carbone 2008; Bahir *et al.* 2009). If this is true, the mismatch between the 68 mol% DNA GC content of *M. smegmatis* and many of the mycobacteriophages is inconsistent. Only phages from the B and K clusters have DNA GC contents this high (Table S3). If the other *Mycobacteria* (other than *M.* *leprae*) are considered, they have DNA GC contents ranging from 65 mol% to 69 mol% (Table S2). Mycobacteriophage clusters that fall within this range include B, C, G, K, I, N, O and P (Table S3). All other clusters fall below this threshold, including sub-60 mol% DNA GC content clusters D, L and H.

The differences between the DNA GC content of isolation host *M. smegmatis* and the DNA GC contents of many phage clusters may indicate that *M. smegmatis* is not the preferred host of many of these phages. Given the tremendous diversity of microbes in the soil (Fierer & Jackson, 2006), it is likely that soils contain numerous permissive hosts for a given phage type, and that phages are able to shift from one host to another as host populations wax and wane. It may be that many mycobacteriophages isolated on *M. smegmatis* actually prefer other close relatives of *M. smegmatis* which also contain high (but not quite as high) DNA GC contents, such as *Corynebacteria* (53.5 mol%), *Rhodococcus* and *Gordonia*.

Expanding upon the previous work of Hatfull and colleagues, it is evident that across the mycobacteriophages discovered to date, there is considerable variation in the DNA GC percentage from cluster to cluster and between subclusters, but little variation between phages belonging to the same subcluster (Jacobs-Sera *et al.*, 2012). Using the A cluster as an example, an analysis of variance of DNA GC content with subcluster as a factor revealed significant differences among subclusters [degrees of freedom (DF)=11 409; *P*<0.0001, F=555.4]. These findings tend to support the idea that subcluster-level differentiation represents rapid diversification and host or niche specialization.

### Encoding tRNAs and codon bias

Of our selected 199 mycobacteriophages, 88 encode genes for tRNAs (Fig. 1, Table S1). The frequency of tRNA genes encoded by the mycobacteriophages varies considerably. Most mycobacteriophage encode zero tRNA genes, but C cluster phage Myrna encodes 41 tRNA genes (Fig. 1, Table S1). In general, the C cluster phages encode the most tRNA genes, which is expected given that they also possess the largest genomes among the mycobacteriophages. A one-way ANOVA of the number of tRNA genes against genome size was highly significant (F=774.9, *P*<0.0001, DF=1198; Fig. 1). A similar result has been reported by Bailly-Bechet and colleagues in their analysis of bacteriophages infecting a wide variety of host types (Bailly-Bechet *et al.*, 2007).

It may be that phages with larger capsids are able to incorporate greater numbers of tRNA genes because space constraints are less stringent. Perhaps larger genomes experience reduced deletional bias that is often characteristic of bacteriophage genomes (Mira *et al.*, 2001; Lawrence *et al.*, 2001). An observation that may have some bearing on this issue is the fact that despite having similar sized genomes, the C cluster phages vary tremendously in the number of tRNA genes they encode.

SCUOs for each genome and gene within the genome were determined using INCA v2.1. Phages that encode a large number of genes for tRNAs were found to also have high SCUO values, indicating a high codon usage bias within that phage genome, as predicted (Fig. 2). Similar correlations between genome codon bias and the presence of tRNA genes have been found for prasinoviruses (Michely *et al.*, 2013), coliphages (Chithambaram *et al.*, 2014) and mimiviruses (Colson *et al.*, 2013). Remarkably, phages that do not possess any genes for tRNAs can exhibit SCUO values just as high as phages that do possess tRNAs (Fig. 2). This finding indicates that these phages have preferred hosts with similar biases for efficient protein translation.

### UPGMA clustering of the mycobacteriophages and their potential hosts

**Fig. 3.**
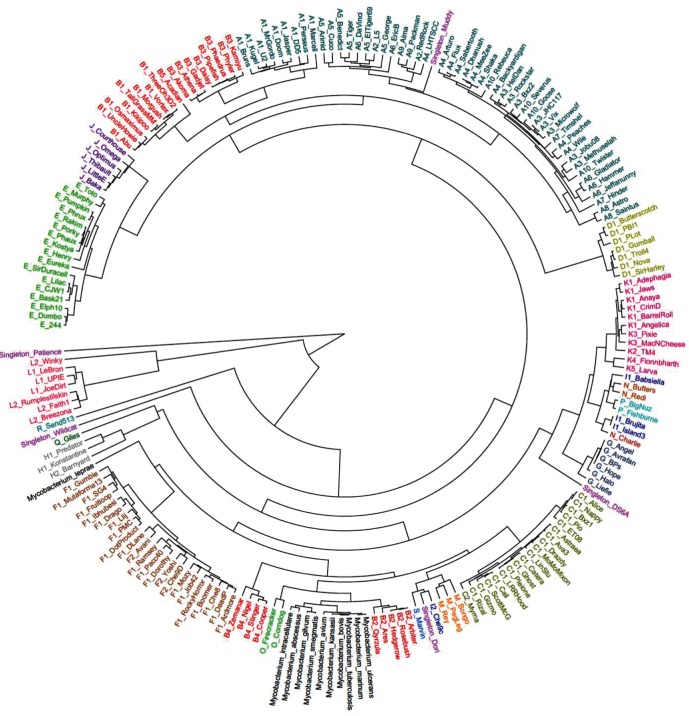
Relative synonymous codon usage (RSCU) was calculated for each mycobacteriophage and mycobacterial species and used to reconstruct this UPGMA-dendrogram. Colors correspond to the cluster designations assigned by phagesdb.org.

We used UPGMA cluster analysis to characterize the frequency of codon usage in mycobacteriophages and to group mycobacteriophage and mycobacterial genomes and proteins based on their RSCU values. A 59-dimensional comparison was performed using the RSCU values for each codon (excluding stop codons, methionine and tryptophan) for a given mycobacteriophage or *Mycobacterium* (Fig. 3). Dendrograms were reconstructed using the UPGMA method to reconstruct UPGMA clusters based on the Pearson correlation between the RSCU values of each codon for each genome (Garcia-Vallvé *et al.*, 1999). As predicted, mycobacteriophages belonging to the same genomic cluster generally shared a UPGMA cluster, but this was not the case for all genomes analyzed. The B and I clusters stand out for being split between multiple, widely separated, branches of the UPGMA dendrogram (Fig. 3). However, mycobacteriophages belonging to the same subcluster possessed strong similarities in codon bias, and this is most notable throughout the B cluster phages, despite their being split among different UPGMA nodes.

Genomic subclusters B1, B3 and B5 are split between branches stemming from a shared UPGMA node with the A and J clusters, highlighting their distinct codon usage bias between the B cluster. The B2 subcluster is found on a branch that shares a UPGMA node with the C, M and O clusters and all but one of the Mycobacteria. This is significant because the B2 phages have been known to infect *M. tuberculosis*. The B4 subcluster is split off from one step above this node. The B3 subcluster is of particular interest because, based upon BlastN alignments, we see regions of high and low similarity with the A1, J and O clusters, which may indicate recombination with phages of these clusters. Nonetheless, recombination does not explain the divergent UPGMA clustering. Instead, it may be the case that adaptation to different host types resulted in similar nucleotide sequences, but distinct codon usage patterns, among the B cluster phages.

On the other hand, the smaller cluster I is split between: (1) the I1 subcluster, which shares a UPGMA node with N and P clusters; and (2) the sole I2 phage, which shares a UPGMA node with the Singleton Dori on a widely separate branch. Analysis of the BlastN alignments suggests that the I2 phage does not share a recent evolutionary history with the I1 subcluster, and the main reason for their sharing the same cluster is a recombination event between the progenitors of the two groups.

The clustering based on RSCU values reveals another dimension of diversity within the mycobacteriophage population (Fig. 3). Across all of the genomes analyzed, three distinct groups emerge after UPGMA analysis: a lone group containing Patience; a second group containing all of the mycobacteriophages belonging to the L genomic cluster; and the rest of the mycobacteriophages, which share a third group. Within the largest UPGMA-cluster, the *Mycobacteria* cluster, with the exception of *M. leprae. Mycobacteria* that are closely related phylogenetically, such as *M. tuberculosis* and *M. bovis*, share a UPGMA cluster on close connecting branches (Gao & Gupta, 2012; Tortoli, 2012). One compelling observation is that *M. leprae*, the most distant relation of the *Mycobacteria*, shares a node with the F cluster phages. Based on this finding, we speculate that there is an evolutionary history of infection among the F cluster phages and *M. leprae*. However, generally speaking, the lack of correlations between mycobacteriophage and mycobacterial host DNA GC content and codon usage patterns suggests that *M. smegmatis* is not the preferred host for these mycobacteriophages.

The A cluster is the largest mycobacteriophage cluster, containing approximately 249 phages (Pope *et al.*, 2011; Jacobs-Sera *et al.*, 2012). In our UPGMA analysis, the A cluster phages appear to be divided among three distinct branches. The first branch harbors only A1-phages and shares a node with some of the B cluster mycobacteriophages. The second and third branches of the A cluster mycobacteriophages share a common node, but are separated throughout the branches in a way that would not be predicted when looking at average nucleotide identities alone. Specifically, the A6 subcluster is found on the two UPGMA branches of the A cluster. It would appear that the A cluster phages share a high degree of nucleotide similarity, but do not subcluster together as distinctly as other mycobacteriophage clusters, reflecting the fact that genomic clusterization of the mycobacteriophages is mainly a useful organizational scheme rather than an attribute with a strong biological basis. In the long run, phylogenetic analysis of the mycobacteriophages, and other phages, may be most effective at the level of the gene.

### Mycobacterium leprae and the mycobacteriophages of the F cluster

*Mycobacterium leprae* is the causative agent of leprosy or Hansen’s disease, and has a long history of infecting humans (Bhat & Prakash, 2012). As such, it is significant that the F cluster mycobacteriophages share a similar codon bias pattern with *M. leprae* (Fig. 3). We conducted an analysis of variance (ANOVA) on the hierarchical UPGMA clustering of RSCU values of each codon among the 23 F cluster phages and *M. leprae*, and found no significant differences among groups. Only the utilization of codons encoding leucine, isoleucine, valine and asparagine showed significantly different RSCU values between the F cluster phages and *M. leprae*. Also differences in the RSCU values for the codons of these amino acids is not uniform across all of the phages – most different are Boomer, Che8, Che9D, SG4 and RockyHorror mycobacteriophages. Moreover, there have been no reported encoded tRNA-genes in the F cluster phages. Although carrying out an infection-assay with the F cluster phages and *M. leprae* TN would be difficult, it is plausible that these phages would be able to infect *M. leprae* in the wild. These could be linked to the ability of the F cluster phages to infect *M. smegmatis*, and thus an adaptation for the host-range of these phages. It would be interesting to see if this is found for other members of the *Mycobacteria* genera.

### The singleton mycobacteriophages and host range expansion

The mycobacteriophage Patience, which is the most genetically distinct mycobacteriophage (Table S1) (Pope *et al.*, 2014), is found at the base of the dendrogram as the sole member of a branch separate from all other mycobacteriophages and *Mycobacteria* (Fig. 3). Compared with the other singleton phages, the singleton Patience is essentially the ‘singleton of the singletons’, although since the time of analysis another closely related phage (Madruga) has been discovered. Given that Patience has the lowest DNA GC content (50.4 mol%) of any phage in this study, it is tempting to speculate that Patience formerly infected a host with a lower DNA GC content, and has recently emerged in *Mycobacteria* (Pope *et al.*, 2014; Dennehy, 2009). Despite the mismatch in DNA GC content between Patience and *M. smegmatis* (68 mol%), Patience does not seem to suffer impaired growth on *M. smegmatis* (Pope *et al.*, 2014; Hatfull, 2015). While Patience's robust growth on *M. smegmatis* seems to imply that differences in codon utilization do not hinder Patience's growth, we note that this growth is achieved under relatively benign laboratory conditions in high-nutrient media. It may be that under more challenging conditions, Patience would be unable to reproduce to high levels because of inefficient translation.

Moreover, Pope *et al.* (2014) point out that Patience does experience codon selection, which is shown by the robust positive correlation between codon selection (adaptive codon enrichment) and the level of gene expression. Finally, a considerable fraction (29 out of 109) of the predicted ORFs were not observed to express peptide products. It is possible that these observations stem from translational failures, although there is no direct evidence to support this.

Another noteworthy singleton, Muddy, shares a similar codon usage pattern with mycobacteriophages belonging to a distinct subset of genomic subclusters within the A cluster (Fig. 3). Interestingly, Muddy is 93.2 % identical at the nucleotide level (E value=0.0) to the phage vB_MapS_FF47, which was isolated from bovine feces using the bacterium *Mycobacterium avium* subspecies *paratuberculosis* ATCC 19698 (Basra *et al.*, 2014). Although it was isolated on *M. avium* ATCC 19698, FF47 was not able to infect six out of eight *M. avium* strains tested, but was able to infect *M. smegmatis* mc^2^155 (Basra *et al.*, 2014). Despite their high degree of similarity, phages FF47 and Muddy were isolated from locations that are approximately 13 890 kilometers apart, Durban, South Africa and Guelph, Canada, respectively (Basra *et al.*, 2014). While Muddy shares an UPGMA node with the A cluster phages, it shares little nucleotide sequence similarity with these phages. We interpret this result as indicating that while Muddy and the A cluster phages do not share an evolutionary history, and hence have little shared sequence identity, they do share similar codon usage patterns due to similar selective pressure, most likely a shared host.

Singleton mycobacteriophage DS6A is the only known mycobacteriophage to infect only mycobacteria of the TB complex (*M. tuberculosis*, *M. bovis*, *M. africanum*, etc.) (Jacobs-Sera *et al.*, 2012; Hatfull *et al.*, 2010). On analysis of the RSCU values, we find that this phage branches off a larger branch containing phages belonging to the G, N, I, P and K clusters. Intriguingly, only mycobacteriophages of the A1, A2, A3, A9, B1, B2, G, K and M subclusters are able to infect *M. tuberculosis* at relatively high efficiencies of plating (i.e. >10^−4^) (Jacobs-Sera *et al.*, 2012; Sampson *et al.*, 2009). The observation that the G and K cluster mycobacteriophages share a UPGMA node with the Singleton phage DS6A is suggestive that there is an underlying genetic similarity between these mycobacteriophages, which enables them to infect *M. tuberculosis*. We speculate that these mycobacteriophages with high-efficiency of *M. tuberculosis* plating contain the requisite gene(s) permitting infection of *M. tuberculosis*, or can easily acquire mutations allowing infection of *M. tuberculosis*. Curiously, the I cluster mycobacteriophages, which shares a subnode of the G cluster with the K, N and P cluster phages, are unable to infect TB Complex bacteria. Presumably, in the past, the I cluster phages possessed the ability to infect *M. tuberculosis,* but have since lost that ability due to one or more mutations (Jacobs-Sera *et al.*, 2012). To our knowledge, P and N cluster phages have not been tested on *M. tuberculosis*, but it would be interesting to see if these phages have the ability to infect this specie﻿

Based on the UPGMA clustering we would not expect the A1, A2, A3, A9, B1, B2 and M cluster phages to be able to infect *M. tuberculosis,* but they can. We speculate that either the common ancestor of these mycobacteriophage clusters possessed the ability to infect *M. tuberculosis* but it was subsequently lost in several diversifying lineages or that these clusters acquired the ability to infect *M. tuberculosis* through mutation or horizontal gene transfer.

### Conclusions

The SEA-PHAGES program has made is possible to conduct large-scale comparative genomic studies of mycobacteriophages. Such a vast collection of sequences allows for large-scale comparative genomic studies that aim to account for the high genetic diversity, dynamic nature and mosaicism of these phages. Codon usage bias is one way of understanding this. Despite cluster organization not representing phylogenetic groupings, we were able to combine the knowledge of cluster assignment with codon usage in order to make inferences about mycobacteriophage host range.

The similarities of codon bias profiles in the mycobacteriophages sheds light on their ability to infect *Mycobacteria*. Our analysis of the mycobacteriophage genomes suggests that, due to the lack of similarities in the DNA GC contents and codon utilization patterns among many mycobacteriophages, the preferred host of many mycobacteriophages is not *M. smegmatis*, despite their having been isolated on *M. smegmatis*.

Further investigation into the structural similarities between DS6A, the only mycobacteriophage cultivated from *M. tuberculosis*, with the mycobacteriophage of the G, I, K, L, N and P clusters may allow identification of mechanisms of *M. tuberculosis* infection, such as host attachment proteins, host receptors and host-specific adaptations. Understanding the mechanisms of bacteriophage infectivity is a necessary step in using these phages therapeutically against *M. tuberculosis*.

## Supplementary Data

78 Supplementary File 1
