# Supplementary material for: Evolutionary interpretations of mycobacteriophage biodiversity and host-range through the analysis of codon usage bias
Source: Microb Genom. 2016 Oct 21;2(10):e000079. doi: 10.1099/mgen.0.000079 (PMC5359403; doi:10.1099/mgen.0.000079)
Supplement: Supplementary File 1 [file mgen-02-79-s001.docx]

**Supplementary Tables for Esposito et al. Evolutionary Interpretations of Mycobacteriophage Biodiversity and Host-Range through the Analysis of Codon Usage Bias**

**Supplementary Table 1.** List of all Mycobacteriophages used in this study.

| Mycobacteriophage | GenBank Accession | Cluster/ Subcluster | Genes | Genome Size (kb) | GC % | SCUO | Encoded  tRNAs | Citations |
| --- | --- | --- | --- | --- | --- | --- | --- | --- |
| Bruns | JN698998 | A/A1 | 94 | 49 | 63.6 | 0.448 | 0 | Pope et al., 2015 |
| DD5 | EU744252 | A/A1 | 87 | 48 | 63.4 | 0.416 | 0 | Hatfull et al., 2010 |
| Doom | JN153085 | A/A1 | 83 | 47 | 63.8 | 0.442 | 0 | Pope et al., 2015 |
| Jasper | EU744251 | A/A1 | 94 | 48 | 63.7 | 0.445 | 0 | Hatfull et al., 2010 |
| JC27 | JF937099 | A/A1 | 97 | 48 | 63.6 | 0.446 | 0 | Pope et al., 2015 |
| Kugel | JN699016 | A/A1 | 90 | 49 | 63.8 | 0.441 | 0 | Pope et al., 2015 |
| Marcell | JX307705 | A/A1 | 82 | 46 | 64.0 | 0.447 | 0 | Pope et al., 2015 |
| MrGordo | JN020140 | A/A1 | 90 | 48 | 63.8 | 0.440 | 0 | Pope et al., 2015 |
| Perseus | JN572689 | A/A1 | 92 | 49 | 63.7 | 0.441 | 0 | Pope et al., 2015 |
| U2 | AY500152 | A/A1 | 81 | 46 | 63.7 | 0.438 | 0 | Hatfull et al., 2010 |
| RedRock | GU339467 | A/A2 | 95 | 49 | 64.5 | 0.482 | 1 | Jacobs-Sera et al., 2010 |
| Trixie | JN408461 | A/A2 | 91 | 49 | 64.5 | 0.479 | 2 | Pope et al., 2015 |
| Bxz2 | AY129332 | A/A3 | 86 | 46 | 64.2 | 0.488 | 3 | Hatfull et al., 2010 |
| HelDan | JF957058 | A/A3 | 86 | 47 | 64.0 | 0.477 | 1 | Pope et al., 2015 |
| JHC117 | JF704098 | A/A3 | 85 | 46 | 64.0 | 0.480 | 3 | Pope et al., 2015 |
| Jobu08 | KC661281 | A/A3 | 85 | 46 | 64.0 | 0.473 | 3 | Pope et al., 2015 |
| Methuselah | KC661272 | A/A3 | 82 | 46 | 64.2 | 0.474 | 0 | Pope et al., 2015 |
| Microwolf | JF704101 | A/A3 | 84 | 46 | 64.0 | 0.479 | 3 | Pope et al., 2015 |
| Rockstar | JF704111 | A/A3 | 76 | 44 | 64.3 | 0.363 | 1 | Pope et al., 2015 |
| Vix | JF704114 | A/A3 | 82 | 46 | 64.0 | 0.478 | 3 | Pope et al., 2015 |
| Arturo | JX307702 | A/A4 | 85 | 48 | 64.0 | 0.457 | 0 | Pope et al., 2015 |
| Backyardigan | JF704093 | A/A4 | 84 | 48 | 63.7 | 0.448 | 0 | Hatfull et al., 2012 |
| Dhanush | KC661271 | A/A4 | 88 | 48 | 63.9 | 0.458 | 0 | Pope et al., 2015 |
| Flux | JQ809701 | A/A4 | 89 | 48 | 63.9 | 0.454 | 0 | Pope et al., 2015 |
| LHTSCC | JN699015 | A/A4 | 89 | 48 | 63.9 | 0.460 | 0 | Hatfull et al., 2012 |
| MeeZee | JN243856 | A/A4 | 87 | 48 | 63.9 | 0.457 | 0 | Hatfull et al., 2012 |
| Peaches | GQ303263 | A/A4 | 86 | 48 | 63.9 | 0.458 | 0 | Pope et al., 2011 |
| Sabertooth | JX307703 | A/A4 | 87 | 48 | 63.9 | 0.457 | 0 | Pope et al., 2015 |
| Shaka | JF792674 | A/A4 | 86 | 48 | 63.9 | 0.459 | 0 | Hatfull et al., 2012 |
| Wile | JN243857 | A/A4 | 82 | 48 | 63.7 | 0.451 | 0 | Hatfull et al., 2012 |
| Benedict | JN083852 | A/A5 | 90 | 47 | 59.8 | 0.377 | 1 | Hatfull et al., 2012 |
| Cuco | JN408459 | A/A5 | 82 | 47 | 60.9 | 0.385 | 1 | Hatfull et al., 2012 |
| ElTiger69 | JX042578 | A/A5 | 91 | 48 | 59.8 | 0.370 | 0 | Hatfull et al., 2013 |
| George | JF704107 | A/A5 | 75 | 46 | 61.0 | 0.394 | 1 | Hatfull et al., 2012 |
| Tiger | JQ684677 | A/A5 | 85 | 46 | 60.7 | 0.389 | 3 | Hatfull et al., 2013 |
| Blue7 | JN698999 | A/A6 | 103 | 48 | 61.4 | 0.430 | 3 | Hatfull et al., 2012 |
| DaVinci | JF937092 | A/A6 | 97 | 46 | 61.5 | 0.437 | 3 | Hatfull et al., 2012 |
| EricB | JN049605 | A/A6 | 97 | 47 | 61.5 | 0.435 | 3 | Hatfull et al., 2012 |
| Gladiator | JF704097 | A/A6 | 95 | 48 | 61.5 | 0.415 | 3 | Hatfull et al., 2012 |
| Hammer | JF937094 | A/A6 | 99 | 49 | 61.3 | 0.422 | 3 | Hatfull et al., 2012 |
| Jeffabunny | JN699019 | A/A6 | 92 | 45 | 61.6 | 0.419 | 3 | Hatfull et al., 2012 |
| HINdeR | KC661275 | A/A7 | 84 | 49 | 62.8 | 0.407 | 0 | Hatfull et al., 2013 |
| Timshel | JF957060 | A/A7 | 85 | 50 | 63.1 | 0.410 | 1 | Hatfull et al., 2012 |
| Astro | JX015524 | A/A8 | 98 | 49 | 61.4 | 0.429 | 0 | Hatfull et al., 2013 |
| Saintus | JN831654 | A/A8 | 93 | 46 | 61.2 | 0.434 | 1 | Hatfull et al., 2012 |
| Alma | JN699005 | A/A9 | 96 | 49 | 62.5 | 0.433 | 1 | Hatfull et al., 2012 |
| PackMan | JF704110 | A/A9 | 91 | 47 | 62.6 | 0.463 | 1 | Hatfull et al., 2012 |
| Goose | JX307704 | A/A10 | 85 | 47 | 65.1 | 0.490 | 0 | Hatfull et al., 2013 |
| Rebeuca | JX411619 | A/A10 | 85 | 48 | 65.1 | 0.478 | 0 | Hatfull et al., 2013 |
| Severus | KC661279 | A/A10 | 79 | 45 | 64.4 | 0.464 | 0 | Hatfull et al., 2013 |
| Twister | JQ512844 | A/A10 | 87 | 48 | 65.0 | 0.481 | 1 | Hatfull et al., 2013 |
| ABU | JF704091 | B/B1 | 100 | 64 | 66.5 | 0.427 | 0 | Hatfull et al., 2012 |
| Kikipoo | JN699017 | B/B1 | 104 | 65 | 66.5 | 0.433 | 0 | Hatfull et al., 2012 |
| Morgushi | JN638753 | B/B1 | 98 | 64 | 66.4 | 0.426 | 0 | Hatfull et al., 2012 |
| Osmaximus | JN006064 | B/B1 | 101 | 65 | 66.3 | 0.431 | 0 | Hatfull et al., 2012 |
| TallGRassMM | JN699010 | B/B1 | 100 | 64 | 66.5 | 0.436 | 0 | Hatfull et al., 2012 |
| ThreeOh3D2 | JN699009 | B/B1 | 103 | 65 | 66.5 | 0.431 | 0 | Hatfull et al., 2012 |
| UncleHowie | GQ303266 | B/B1 | 98 | 64 | 66.5 | 0.427 | 0 | Pope, et al., 2011 |
| Vortex | JF704103 | B/B1 | 98 | 64 | 66.5 | 0.424 | 0 | Hatfull et al., 2012 |
| Arbiter | JN618996 | B/B2 | 88 | 63 | 68.9 | 0.504 | 0 | Hatfull et al., 2012 |
| Aries | JN699004 | B/B2 | 92 | 64 | 69.0 | 0.508 | 0 | Hatfull et al., 2012 |
| Hedgerow | JN698991 | B/B2 | 91 | 64 | 69.0 | 0.509 | 0 | Hatfull et al., 2012 |
| Qyrzula | DQ398048 | B/B2 | 81 | 61 | 68.9 | 0.504 | 0 | Hatfull et al., 2010 |
| Rosebush | AY129334 | B/B2 | 90 | 63 | 68.9 | 0.502 | 0 | Hatfull et al., 2010 |
| Akoma | JN699006 | B/B3 | 104 | 65 | 67.5 | 0.509 | 0 | Hatfull et al., 2012 |
| Athena | JN699003 | B/B3 | 104 | 66 | 67.5 | 0.501 | 0 | Hatfull et al., 2012 |
| Daisy | JF704095 | B/B3 | 100 | 65 | 67.6 | 0.503 | 0 | Hatfull et al., 2012 |
| Gadjet | JN698992 | B/B3 | 102 | 65 | 67.5 | 0.504 | 0 | Hatfull et al., 2012 |
| Kamiyu | JN699018 | B/B3 | 103 | 65 | 67.5 | 0.502 | 0 | Hatfull et al., 2012 |
| Phaedrus | EU816589 | B/B3 | 98 | 64 | 67.6 | 0.500 | 0 | Hatfull et al., 2010 |
| Phlyer | FJ641182 | B/B3 | 103 | 66 | 67.5 | 0.502 | 0 | Copeland et al., 2009 |
| Pipefish | DQ398049 | B/B3 | 102 | 65 | 67.3 | 0.482 | 0 | Hatfull et al., 2010 |
| Cooper | DQ398044 | B/B4 | 99 | 67 | 69.1 | 0.545 | 0 | Hatfull et al., 2010 |
| Nigel | EU770221 | B/B4 | 94 | 66 | 68.3 | 0.508 | 1 | Hatfull et al., 2010 |
| Stinger | JN699011 | B/B4 | 95 | 66 | 68.6 | 0.505 | 0 | Hatfull et al., 2012 |
| Zemanar | JF704104 | B/B4 | 94 | 67 | 68.9 | 0.543 | 0 | Hatfull et al., 2012 |
| Acadian | JN699007 | B/B5 | 96 | 66 | 68.4 | 0.478 | 0 | Hatfull et al., 2012 |
| Alice | JF704092 | C/C1 | 221 | 142 | 64.7 | 0.500 | 29 | Hatfull et al., 2012 |
| ArcherS7 | KC748970 | C/C1 | 237 | 144 | 64.7 | 0.490 | 31 | Hatfull et al., 2013 |
| Astraea | KC691257 | C/C1 | 232 | 142 | 64.7 | 0.492 | 31 | Hatfull et al., 2013 |
| Ava3 | JQ911768 | C/C1 | 232 | 142 | 64.8 | 0.498 | 32 | Hatfull et al., 2013 |
| Bxz1 | AY129337 | C/C1 | 225 | 141 | 64.8 | 0.492 | 35 | Hatfull et al., 2010 |
| Cali | EU826471 | C/C1 | 222 | 142 | 64.7 | 0.488 | 35 | Hatfull et al., 2010 |
| Catera | DQ398053 | C/C1 | 218 | 139 | 64.7 | 0.498 | 35 | Hatfull et al., 2010 |
| Dandelion | JN412588 | C/C1 | 241 | 145 | 64.7 | 0.494 | 32 | Hatfull et al., 2012 |
| Drazdys | JF704116 | C/C1 | 215 | 143 | 64.7 | 0.485 | 28 | Bambawale et al., 2012 |
| ET08 | GQ303260 | C/C1 | 221 | 142 | 64.6 | 0.493 | 30 | Pope et al., 2011 |
| Ghost | JF704096 | C/C1 | 231 | 143 | 64.6 | 0.494 | 34 | Hatfull et al., 2012 |
| Gizmo | KC748968 | C/C1 | 241 | 147 | 64.6 | 0.488 | 32 | Hatfull et al., 2013 |
| LinStu | JN412592 | C/C1 | 263 | 141 | 64.8 | 0.499 | 30 | Hatfull et al., 2012 |
| LRRHood | GQ303262 | C/C1 | 227 | 142 | 64.7 | 0.489 | 30 | Pope et al., 2011 |
| MoMoMixon | JN699626 | C/C1 | 229 | 142 | 64.8 | 0.496 | 33 | Hatfull et al., 2012 |
| Nappy | JN699627 | C/C1 | 233 | 144 | 64.7 | 0.494 | 33 | Hatfull et al., 2012 |
| Pio | JN699013 | C/C1 | 238 | 145 | 64.8 | 0.494 | 35 | Hatfull et al., 2012 |
| Pleione | JN624850 | C/C1 | 237 | 144 | 64.7 | 0.497 | 36 | Hatfull et al., 2012 |
| Rizal | EU826467 | C/C1 | 220 | 141 | 64.7 | 0.489 | 35 | Hatfull et al., 2010 |
| ScottMcG | EU826469 | C/C1 | 221 | 140 | 64.8 | 0.500 | 35 | Hatfull et al., 2010 |
| Myrna | EU826466 | C/C2 | 229 | 153 | 65.4 | 0.566 | 41 | Hatfull et al., 2010 |
| Butterscotch | FJ168660 | D/D1 | 86 | 59 | 59.7 | 0.330 | 0 | Hatfull et al., 2010 |
| Gumball | FJ168661 | D/D1 | 88 | 59 | 59.6 | 0.333 | 0 | Hatfull et al., 2010 |
| Nova | JN699014 | D/D1 | 88 | 60 | 59.7 | 0.331 | 0 | Hatfull et al., 2012 |
| PBI1 | DQ398047 | D/D1 | 81 | 57 | 59.8 | 0.331 | 0 | Hatfull et al., 2010 |
| PLot | DQ398051 | D/D1 | 89 | 59 | 59.8 | 0.326 | 0 | Hatfull et al., 2010 |
| SirHarley | JF937107 | D/D1 | 89 | 59 | 59.6 | 0.339 | 0 | Hatfull et al., 2012 |
| Troll4 | FJ168662 | D/D1 | 88 | 59 | 59.6 | 0.327 | 0 | Hatfull et al., 2010 |
| 244 | DQ398041 | E | 142 | 70 | 63.4 | 0.433 | 2 | Hatfull et al., 2010 |
| Bask21 | JF937091 | E | 143 | 69 | 62.9 | 0.450 | 2 | Hatfull et al., 2012 |
| CJW1 | AY129331 | E | 141 | 71 | 63.7 | 0.433 | 2 | Hatfull et al., 2010 |
| Dumbo | KC691255 | E | 147 | 70 | 63.0 | 0.438 | 2 | Hatfull et al., 2013 |
| Elph10 | JN391441 | E | 143 | 70 | 63.0 | 0.437 | 2 | Hatfull et al., 2012 |
| Eureka | JN412590 | E | 145 | 71 | 62.9 | 0.435 | 2 | Hatfull et al., 2012 |
| Henry | JF937096 | E | 145 | 71 | 63.0 | 0.445 | 2 | Hatfull et al., 2012 |
| Kostya | EU816591 | E | 143 | 69 | 63.5 | 0.441 | 2 | Hatfull et al., 2010 |
| Lilac | JN382248 | E | 140 | 70 | 63.0 | 0.433 | 2 | Hatfull et al., 2012 |
| Murphy | KC748971 | E | 145 | 71 | 62.9 | 0.435 | 0 | Hatfull et al., 2013 |
| Phaux | KC748969 | E | 144 | 71 | 62.9 | 0.436 | 0 | Hatfull et al., 2013 |
| Phrux | KC661277 | E | 140 | 70 | 63.1 | 0.438 | 0 | Hatfull et al., 2013 |
| Porky | EU816588 | E | 147 | 70 | 63.5 | 0.440 | 2 | Hatfull et al., 2010 |
| Pumpkin | GQ303265 | E | 143 | 69 | 66.0 | 0.438 | 2 | Pope et al., 2011 |
| Rakim | JN006062 | E | 142 | 70 | 62.9 | 0.440 | 2 | Hatfull et al., 2012 |
| Sir Duracell | JF937106 | E | 150 | 70 | 62.9 | 0.445 | 0 | Hatfull et al., 2012 |
| Toto | JN006061 | E | 135 | 70 | 63.0 | 0.444 | 2 | Hatfull et al., 2012 |
| Ardmore | GU060500 | F/F1 | 88 | 48 | 61.5 | 0.340 | 0 | Henry et al., 2010 |
| Boomer | EU816590 | F/F1 | 105 | 54 | 61.5 | 0.347 | 0 | Hatfull et al., 2010 |
| Che8 | AY129330 | F/F1 | 112 | 56 | 61.3 | 0.347 | 0 | Hatfull et al., 2010 |
| DeadP | JN698996 | F/F1 | 106 | 53 | 61.6 | 0.366 | 0 | Hatfull et al., 2012 |
| Dlane | JF937093 | F/F1 | 105 | 55 | 61.9 | 0.358 | 0 | Hatfull et al., 2012 |
| Dorothy | JX411620 | F/F1 | 103 | 55 | 61.4 | 0.357 | 0 | Hatfull et al., 2013 |
| DotProduct | JN859129 | F/F1 | 95 | 52 | 61.8 | 0.374 | 0 | Hatfull et al., 2012 |
| Drago | JN542517 | F/F1 | 102 | 52 | 61.2 | 0.355 | 0 | Hatfull et al., 2012 |
| Fruitloop | FJ174690 | F/F1 | 102 | 53 | 61.8 | 0.357 | 0 | Hatfull et al., 2010 |
| Gumbie | JN398368 | F/F1 | 103 | 55 | 61.4 | 0.371 | 0 | Hatfull et al., 2012 |
| Ibhubesi | JF937098 | F/F1 | 105 | 53 | 61.2 | 0.350 | 0 | Hatfull et al., 2012 |
| Job42 | KC661280 | F/F1 | 106 | 57 | 61.2 | 0.356 | 0 | Hatfull et al., 2013 |
| Llij | DQ398045 | F/F1 | 100 | 53 | 61.5 | 0.340 | 0 | Hatfull et al., 2010 |
| Mozy | JF937102 | F/F1 | 113 | 54 | 61.1 | 0.344 | 0 | Hatfull et al., 2012 |
| Mutaforma13 | JN020142 | F/F1 | 105 | 54 | 61.3 | 0.363 | 0 | Hatfull et al., 2012 |
| Pacc40 | FJ174692 | F/F1 | 101 | 56 | 61.3 | 0.349 | 0 | Hatfull et al., 2010 |
| PMC | DQ398050 | F/F1 | 104 | 53 | 61.4 | 0.356 | 0 | Hatfull et al., 2010 |
| Ramsey | FJ174693 | F/F1 | 108 | 56 | 61.2 | 0.360 | 0 | Hatfull et al., 2010 |
| RockyHorror | JF704117 | F/F1 | 102 | 54 | 61.1 | 0.487 | 0 | Hatfull et al., 2012 |
| SG4 | JN699012 | F/F1 | 105 | 56 | 61.9 | 0.353 | 0 | Hatfull et al., 2012 |
| Avani | JQ809702 | F/F2 | 107 | 52 | 61.0 | 0.363 | 0 | Hatfull et al., 2013 |
| Che9d | AY129336 | F/F2 | 111 | 53 | 60.9 | 0.369 | 0 | Hatfull et al., 2010 |
| Yoshi | JF704115 | F/F2 | 116 | 56 | 61.0 | 0.359 | 0 | Hatfull et al., 2012 |
| Angel | FJ973624 | G | 61 | 40 | 66.7 | 0.428 | 0 | Sampson et al., 2009 |
| Avrafan | JN699002 | G | 62 | 41 | 66.6 | 0.425 | 0 | Hatfull et al., 2012 |
| BPs | EU568876 | G | 63 | 41 | 66.6 | 0.428 | 0 | Hatfull et al., 2010 |
| Halo | DQ398042 | G | 64 | 41 | 66.7 | 0.436 | 0 | Hatfull et al., 2010 |
| Hope | GQ303261 | G | 63 | 41 | 66.6 | 0.426 | 0 | Pope et al., 2011 |
| Liefie | JN412593 | G | 61 | 40 | 66.8 | 0.437 | 0 | Hatfull et al., 2012 |
| Konstantine | FJ174691 | H/H1 | 95 | 63 | 57.4 | 0.275 | 0 | Hatfull et al., 2010 |
| Predator | EU770222 | H/H1 | 92 | 61 | 56.4 | 0.261 | 0 | Hatfull et al., 2010 |
| Barnyard | AY129339 | H/H2 | 109 | 67 | 57.5 | 0.289 | 0 | Hatfull et al., 2010 |
| Babsiella | JN699001 | I/I1 | 78 | 47 | 67.1 | 0.465 | 0 | Hatfull et al., 2012 |
| Brujita | FJ168659 | I/I1 | 74 | 45 | 66.8 | 0.456 | 0 | Hatfull et al., 2010 |
| Island3 | HM152765 | I/I1 | 76 | 45 | 66.8 | 0.453 | 0 | P Pope et al., 2011 |
| Che9C | AY129333 | I/I2 | 84 | 53 | 65.4 | 0.396 | 0 | Hatfull et al., 2010 |
| Baka | JF937090 | J | 245 | 106 | 60.7 | 0.346 | 1 | Hatfull et al., 2012 |
| Courthouse | JN698997 | J | 241 | 104 | 60.9 | 0.350 | 2 | Hatfull et al., 2012 |
| LittleE | JF937101 | J | 229 | 103 | 61.3 | 0.349 | 1 | Hatfull et al., 2012 |
| Omega | AY129338 | J | 237 | 104 | 61.4 | 0.358 | 2 | Hatfull et al., 2010 |
| Optimus | JF957059 | J | 230 | 103 | 60.8 | 0.343 | 1 | Hatfull et al., 2012 |
| Thibault | JN201525 | J | 216 | 99 | 60.8 | 0.340 | 2 | Hatfull et al., 2012 |
| Adephagia | JF704105 | K/K1 | 94 | 55 | 66.6 | 0.450 | 1 | Alferez, et al., 2012 |
| Anaya | JF704106 | K/K1 | 98 | 56 | 66.4 | 0.440 | 1 | Hatfull et al., 2012 |
| Angelica | HM152764 | K/K1 | 94 | 55 | 66.4 | 0.436 | 1 | Pope et al., 2011 |
| BarrelRoll | JN643714 | K/K1 | 95 | 55 | 66.6 | 0.448 | 1 | Hatfull et al., 2012 |
| CrimD | HM152767 | K/K1 | 96 | 55 | 66.9 | 0.456 | 1 | Pope et al., 2011 |
| JAWS | JN185608 | K/K1 | 94 | 55 | 66.6 | 0.447 | 1 | Hatfull et al., 2012 |
| TM4 | AF068845 | K/K2 | 89 | 50 | 68.1 | 0.516 | 0 | Hatfull et al., 2010 |
| MacnCheese | JX042579 | K/K3 | 99 | 56 | 67.3 | 0.484 | 0 | Hatfull et al., 2013 |
| Pixie | JF937104 | K/K3 | 100 | 56 | 67.3 | 0.482 | 0 | Pope et al., 2015 |
| Fionnbharth | JN831653 | K/K4 | 94 | 55 | 68.0 | 0.506 | 1 | Hatfull et al., 2012 |
| Larva | JN243855 | K/K5 | 96 | 58 | 65.3 | 0.417 | 1 | Hatfull et al., 2012 |
| JoeDirt | JF704108 | L/L1 | 113 | 68 | 58.8 | 0.345 | 9 | Hatfull et al., 2012 |
| LeBron | HM152763 | L/L1 | 120 | 67 | 58.8 | 0.341 | 9 | Pope et al., 2011 |
| UPIE | JF704113 | L/L1 | 122 | 67 | 58.8 | 0.341 | 9 | Hatfull et al., 2012 |
| Breezona | KC691254 | L/L2 | 132 | 69 | 58.9 | 0.364 | 12 | Hatfull et al., 2013 |
| Faith1 | JF744988 | L/L2 | 128 | 68 | 58.9 | 0.368 | 12 | Hatfull et al., 2012 |
| Rumpelstiltskin | JN680858 | L/L2 | 106 | 63 | 58.9 | 0.340 | 1 | Hatfull et al., 2012 |
| Winky | KC661276 | L/L2 | 131 | 69 | 58.9 | 0.363 | 0 | Hatfull et al., 2013 |
| Bongo | JN699628 | M | 132 | 68 | 61.6 | 0.441 | 19 | Hatfull et al., 2012 |
| PegLeg | KC900379 | M | 139 | 69 | 61.5 | 0.441 | 16 | Hatfull et al., 2013 |
| Rey | JF937105 | M | 153 | 72 | 60.9 | 0.441 | 21 | Hatfull et al., 2012 |
| Butters | KC576783 | N | 66 | 39 | 65.8 | 0.406 | 0 | Hatfull et al., 2012 |
| Charlie | JN256079 | N | 63 | 41 | 66.3 | 0.423 | 0 | Hatfull et al., 2012 |
| Redi | JN624851 | N | 68 | 41 | 66.1 | 0.416 | 0 | Hatfull et al., 2012 |
| Corndog | AY129335 | O | 99 | 65 | 65.4 | 0.453 | 0 | Hatfull et al., 2010 |
| Firecracker | JN698993 | O | 128 | 67 | 65.5 | 0.469 | 0 | Hatfull et al., 2012 |
| BigNuz | JN412591 | P | 82 | 47 | 66.7 | 0.436 | 0 | Hatfull et al., 2012 |
| Fishburne | KC691256 | P | 77 | 45 | 67.3 | 0.452 | 0 | Hatfull et al., 2013 |
| Giles | EU203571 | Q | 78 | 49 | 67.3 | 0.446 | 0 | Hatfull et al., 2010 |
| Send513 | JF704112 | R | 96 | 66 | 56.0 | 0.278 | 0 | Hatfull et al., 2012 |
| Marvin | JF704100 | S | 107 | 60 | 63.4 | 0.417 | 0 | Hatfull et al., 2012 |
| Dori | JN698995 | Singleton | 93 | 60 | 66.0 | 0.357 | 0 | Hatfull et al., 2012 |
| DS6A | JN698994 | Singleton | 97 | 56 | 68.4 | 0.100 | 0 | Hatfull et al., 2012 |
| Muddy | KF024728 | Singleton | 71 | 47 | 58.8 | 0.052 | 0 | Hatfull et al., 2013 |
| Patience | JN412589 | Singleton | 108 | 66 | 50.0 | 0.023 | 1 | Hatfull et al., 2012 |
| Wildcat | DQ398052 | Singleton | 147 | 70 | 57.0 | 0.023 | 24 | Hatfull et al., 2010 |

**Supplementary Table 2.** List of all Mycobacterial species used.

| Mycobacterial Strain | GenBank Accession | Genes | Genome Size (kb) | GC % | Citation |
| --- | --- | --- | --- | --- | --- |
| *M. abscessus* *bolletii* 50594 | CP004374 | 4957 | 4618 | 65 | Kim et al., 2103 |
| *M. avium* K-10 | NC_002944 | 3935 | 3981 | 69.0 | Li et al., 2005 |
| *M. bovis* BCG Pasteur 1173P2 | NC_008769 | 3952 | 3972 | 66 | Brosch et al., 2007 |
| *M. gilvum* Spyr1 | NC_014814 | 5139 | 5077 | 68 | Kallimanis et al., 2011 |
| *M. intracellulare* ATCC 13950 | NC_016946 | 5143 | 4936 | 69 | Kim et al., 2012 |
| *M. kansasii* ATCC 12478 | NC_022663 | 5711 | 5712 | 67 | Veyrier & Behr 2013 |
| *M. leprae* TN | NC_002677 | 1604 | 1620 | 57.8 | Cole et al., 2001 |
| *M. marinum* M | NC_010612 | 5422 | 5973 | 66 | Stinear et al., 2008 |
| *M. smegmatis* MC2 155 | NC_018289 | 6692 | 6508 | 68 | Gallien et al., 2010 |
| *M. tuberculosis* H37Rv | NC_000962 | 3935 | 3981 | 65.6 | Cole et al., 2001 |
| *M. ulcerans* Agy99 | NC_005916 | 4159 | 4074 | 66 | Stinear et al., 2004 |

**Supplementary Table 3** - Mean GC content for each mycobacteriophage subcluster analyzed in this work.

| Subcluster | Mean GC Content |
| --- | --- |
| H1 | 57.5 |
| H2 | 57.5 |
| L1 | 58.8 |
| L2 | 58.8929 |
| D1 | 59.71 |
| A5 | 60.5481 |
| J | 60.9091 |
| F2 | 61.02 |
| A8 | 61.3667 |
| A6 | 61.4792 |
| F1 | 61.499 |
| M | 61.6 |
| A9 | 62.54 |
| E | 63.0203 |
| A7 | 63.1 |
| A2 | 63.3783 |
| A1 | 63.6771 |
| A4 | 63.8943 |
| A3 | 64.0329 |
| C1 | 64.7052 |
| A10 | 64.9 |
| K5 | 65.09 |
| C2 | 65.3 |
| O | 65.4143 |
| I2 | 65.5 |
| N | 66.1538 |
| B1 | 66.4723 |
| G1 | 66.6111 |
| I1 | 66.9 |
| K1 | 66.9375 |
| P | 67.125 |
| K3 | 67.3 |
| B3 | 67.4895 |
| K4 | 67.85 |
| B5 | 68.0333 |
| K2 | 68.2714 |
| B4 | 68.8455 |
| B2 | 68.95 |

**Supplementary Table 4.** Overall relative synonymous codon usage for all 199 mycobacteriophages was calculated. Asterisk (*) denotes the preferred codon with higher RSCU value.

| Amino Acid | Codon | RSCU | Amino Acid | Codon | RSCU |
| --- | --- | --- | --- | --- | --- |
| Thr | ACA | 0.717259615 | **Cys** | UGU | 0.746730769 |
|  | ACG * | 1.374519231 |  | UGC * | 1.25326923 |
|  | ACC | 1.34788462 | **Tyr** | UAU | 0.502980769 |
|  | ACU | 0.560625 |  | UAC * | 1.497019231 |
| Ser | AGC * | 1.128221154 | **Gly** | GGU | 0.916778846 |
|  | AGU | 0.533028846 |  | GGG | 0.830913462 |
| Ile | AUU | 0.586105769 |  | GGC * | 1.487451923 |
|  | AUC * | 2.107836538 |  | GGA | 0.765048077 |
|  | AUA | 0.305769231 | **Ala** | GCU | 0.758461538 |
| Met | AUG | 1 |  | GCG | 1.310961538 |
| Pro | CCA | 0.816971154 |  | GCC * | 1.140336538 |
|  | CCC | 0.895961538 |  | GCA | 0.789855769 |
|  | CCG * | 1.578653846 | **Asp** | GAU | 0.776442308 |
|  | CCU | 0.708942308 |  | GAC * | 1.223557692 |
| Leu | CUA | 0.526153846 | **Glu** | GAG | 1.137019231 |
|  | CUC | 1.641105769 |  | GAA | 0.862980769 |
|  | CUG * | 1.962403846 | **Arg** | CGU | 0.827451923 |
|  | UUA | 0.176826923 |  | CGG | 1.396057692 |
|  | UUG * | 0.88 |  | AGG * | 0.688605769 |
| Val | GUA | 0.492211538 |  | AGA | 0.463413462 |
|  | GUC * | 1.406442308 |  | CGA | 1.257548077 |
|  | GUG | 1.312115385 | **His** | CAU | 0.779086538 |
|  | GUU | 0.788990385 |  | CAC * | 1.220913462 |
| Ser | UCA | 0.905096154 | **Gln** | CAG * | 1.138413462 |
|  | UCC | 1.022740385 |  | CAA | 0.861586538 |
|  | UCG * | 1.791875 | **Asn** | AAU | 0.486298077 |
|  | UCU | 0.618798077 |  | AAC * | 1.513798077 |
| Trp | UGG | 1 | **Lys** | AAG * | 1.497596154 |
| Phe | UUU | 0.437644231 |  | AAA | 0.502451923 |
|  | UUC * | 1.562355769 |  |  |  |
